# Untargeted serum metabolomics reveals potential biomarkers and metabolic pathways associated with esophageal cancer

**DOI:** 10.3389/fonc.2022.938234

**Published:** 2022-09-13

**Authors:** Xiao-li Yang, Peng Wang, Hua Ye, Ming Jiang, Yu-bin Su, Xuan-xian Peng, Hui Li, Jian-ying Zhang

**Affiliations:** ^1^ State Key Laboratory of Esophageal Cancer Prevention & Treatment, Zhengzhou University, Zhengzhou, China; ^2^ State Key Laboratory of Bio-Control, School of Life Sciences, Southern Marine Science and Engineering Guangdong Laboratory (Zhuhai), Sun Yat-sen University, University City, Guangzhou, China; ^3^ Henan Key Laboratory of Tumor Epidemiology and College of Public Health, Zhengzhou University, Zhengzhou, China; ^4^ Key Laboratory of Functional Protein Research of Guangdong Higher Education Institutes, Department of Biotechnology, College of Life Science and Technology, Jinan University, Guangzhou, China; ^5^ Henan Academy of Medical and Pharmaceutical Sciences, Zhengzhou University, Zhengzhou, China

**Keywords:** esophageal cancer, metabolomics, biomarker, malignant degrees, therapeutic efficacy

## Abstract

Metabolomics has been reported as an efficient tool to screen biomarkers that are related to esophageal cancer. However, the metabolic biomarkers identifying malignant degrees and therapeutic efficacy are still largely unknown in the disease. Here, GC-MS-based metabolomics was used to understand metabolic alteration in 137 serum specimens from patients with esophageal cancer, which is approximately two- to fivefold as many plasma specimens as the previous reports. The elevated amino acid metabolism is in sharp contrast to the reduced carbohydrate as a characteristic feature of esophageal cancer. Comparative metabolomics showed that most metabolic differences were determined between the early stage (0–II) and the late stage (III and IV) among the 0–IV stages of esophageal cancer and between patients who received treatment and those who did not receive treatment. Glycine, serine, and threonine metabolism and glycine were identified as the potentially overlapped metabolic pathway and metabolite, respectively, in both disease progress and treatment effect. Glycine, fructose, ornithine, and threonine can be a potential array for the evaluation of disease prognosis and therapy in esophageal cancer. These results highlight the means of identifying previously unknown biomarkers related to esophageal cancer by a metabolomics approach.

## Introduction

Esophageal cancer is a common malignant gastrointestinal tumor that ranks seventh and sixth in global cancer incidence and mortality, respectively. The cancer is known for typical geographic distribution in incidence and poor prognosis (1, 2). According to the data released by the International Agency for Research on Cancer (IARC), there were 604,100 new cases of esophageal cancer and 544,076 deaths due to the disease worldwide in 2020, with China accounting for 53.7% and 55.4%, respectively (3). Notably, it is predicted that the incidence and mortality of esophageal cancer will rise yearly owing to the increasing aging of the population, posing a major challenge for health practitioners and a huge threat to human health (4). Thus, understanding the pathogenesis of esophageal cancer is helpful to identify new biomarkers for disease progression judgment and treatment prognosis.

Esophageal cancer is generally divided into two subtypes, namely, esophageal squamous cell carcinoma (ESCC) and esophageal adenocarcinoma (EAC); most esophageal cancers are ESCC. ESCC carcinogenesis is related to external and internal factors and, thus, is a multifactorial disease. External factors include tobacco, alcohol, and behavior at high temperature (5–7), while internal factors are attributed to molecular events. However, the precise molecular events underlying ESCC etiology are only partially understood and thereby the detailed mechanism of occurrence and progression of ESCC has not been holistically revealed yet (8, 9). These cause limited targeted therapies and insufficient clinical management in ESCC patients. Therefore, further understanding the pathogenesis of esophageal cancer and identifying diagnosis biomarkers can markedly improve the prognosis and therapy of patients with esophageal cancer.

Recently developed metabolomics provides an efficient approach to achieve a global assessment and validation of endogenous small-molecule metabolites within a cell or biologic system including cancer samples (10, 11). Metabolomics is a key tool for biomarker discovery and personalized medicine including cancers (12, 13). This leads to the identification of quantitative metabolic biomarkers for cancer detection and/or assessment of efficacy of anticancer treatment. Yang et al. adopt liquid chromatography with tandem mass spectrometry (LC-MS/MS)-based metabolomics to identify biomarkers in 60 postoperative esophageal tissues compared with 15 normal tissues adjacent to the tumor and indicate that glycerophospholipid metabolism is associated with the ESCC tumorigenesis and progression (14). Zang et al. use spatially resolved metabolomics to discover cancer tissue-relevant metabolic signatures and identify glutamine metabolism, fatty acid metabolism, *de novo* synthesis phosphatidylcholine, and phosphatidylethanolamine as the metabolic signatures (15). Fujigaki et al. identify serum arabitol, glycine, L-serine, and L-arginine as biomarkers predicting the chemoradiosensitivity of ESCC patients using the targeted metabolomics approach (16). These results indicate that metabolomics is an effective approach to identify biomarkers that are related to esophageal cancer. However, the association between metabolic alterations in serum samples and progression and therapeutic effect of patients with esophageal cancer remains unclear. In particular, how can differentiating post-treatment and pre-treatment patients leads to “optimized treatment” needs further exploration.

Here, gas chromatography–mass spectrometry (GC-MS)-based metabolomics was performed to carry out a metabolomics analysis of 137 serum specimens from patients with esophageal cancer. Comparative metabolomics showed that the patients with esophageal cancer exhibited enhanced amino acid against the reduced carbohydrate as a previously unknown characteristic feature of esophageal cancer. Then, the association of metabolic modulation with malignant degrees of esophageal cancer was investigated. The most significant metabolic difference was detected between stages 0–II and III–IV. Finally, the effect of treatment on metabolism was explored between patients who received treatment and those who did not. The treatment reverted the metabolic state of the patients to close to that of healthy individuals, but further studies are needed to verify them using the same group of patients compared before and after treatment. These results provide a novel insight to further understand the pathogenesis of esophageal cancer and previously unknown biomarkers for prognosis and treatment evaluation of the patients.

## Results

### The detailed clinical characteristics of patients with esophageal cancer

Sera were collected from 137 patients with esophageal cancer, namely, 104 male and 33 female patients with 56 cases ≤60 years and 81 cases >60 years. Of these, 121 patients had ESCC, 1 patient had EAC, and 15 patients had other pathological types. A total of 137 patients were distributed in five stages with 7 in stage 0, 26 in stage I, 27 in stage II, 30 in stage III, and 17 in stage IV. Among them, 30 patients had experienced treatment and 107 were still in the untreated stage. There were no differences in gender between esophageal cancer patients and healthy individuals, and between late stage and early stage, but there was a difference between patients who received treatment and those who did not. There was also no difference in the smoking habit between the comparison groups (**Table 1**). In addition, the population studied favors oriental traditional diets.

**Table 1:** Characteristics of subjects.

Characteristics	Gender			Age		Smoking habit		
	Male (*N*)	Female (*N*)	*p*-value	(years)	*p*-value	Smoke (*N*)	No smoke (*N*)	*p*-value
**Subjects**								
Healthy	19	11	0.157	62.6 ± 6.7	0.859	12	18	0.246
Esophageal cancer	104	33		62.4 ± 9.7		46	42	
**Subjects**								
Healthy	19	11	0.753	62.6 ± 6.7	0.180	12	18	0.739
Early stage	40	20		65.2 ± 9.0		15	19	
**Difference stage**								
Stage 0–II	40	20	0.168	65.2 ± 9.0	0.271	15	19	0.897
Stage III–IV	37	10		63.2 ± 9.4		11	13	
**Treatment**								
Pre-treatment	77	30	0.041	64.3 ± 9.2	3.84E-6	26	32	0.052
Post-treatment	27	3		55.4 ± 8.4		20	10	

### Quality control and metabolite identifications for metabolomics

To ensure basic validation of the instrument for metabolite profiling, quality control (QC) was conducted. We prepared pooled QC samples by equally mixing small aliquots of sera from the samples studied, and one QC sample was run after every 14 samples. Orthogonal partial least-squares discriminant analysis (OPLS-DA) showed that QC samples were tightly clustered, which indicated that the sample analysis sequence had satisfactory stability and repeatability (**Supplementary Figure 1**). Meanwhile, for metabolite identification, 30 pure reference compounds and alkane mix 34 (C7–C40) were analyzed. Two independent parameters (mass spectra and retention index) were recorded and matched. A total of 58 metabolites were identified, where 28 and 18 were located in Metabolomics Standards Initiative (MSI) level 1 and 2, respectively (**Supplementary Table 1**).

### Metabolomic profiling of esophageal cancer patients and healthy individuals

**Figure 1:**
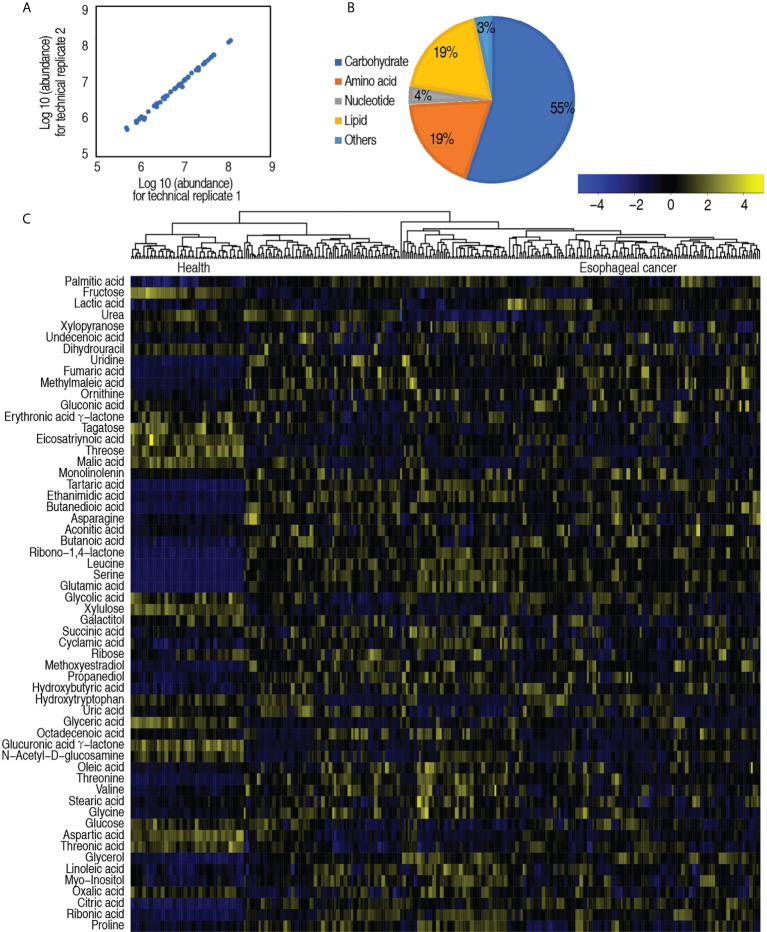
Serum metabolic profile of patients with esophageal cancer. **(A)** Reproducibility of metabolomic profiling platform. Abundance of metabolites quantified in samples over two technical repeats is shown. Correlation coefficient between technical repeats varies between 0.990 and 0.999. **(B)** Categories of the global metabolites. **(C)** Heatmap showing global metabolites. Yellow color and blue color indicate increase and decrease of metabolites relative to the median metabolite level, respectively (see color scale).

To study the metabolic changes of patients with esophageal cancer, a nontargeted metabolic profiling strategy based on GC-MS was applied. A total of 167 serum specimens from 137 esophageal cancer patients and 30 healthy individuals were enrolled in this study. Each individual sample was subjected to two technical repeats, yielding a total of 334 data sets. The correlation coefficient between technical replicates varied between 0.990 and 0.999 (**Figure 1A**), demonstrating the reproducibility of the data. After the removal of internal standard ribitol and any known artificial peaks and the integration of the same compounds, 58 metabolites were identified. These metabolites were categorized into carbohydrate (55%), amino acid (19%), nucleotide (4%), lipid (19%), and others (3%) (**Figure 1B**). The metabolic profiles of patients with esophageal cancer and healthy individuals were displayed as a heatmap (**Figure 1C**), where the two groups were clearly separated. OPLS-DA showed that component t[1] differentiates the two groups (**Supplementary Figure 2**). These results indicate that patients with esophageal cancer have a metabolic shift.

### Differential metabolomic profiling of patients with esophageal cancer compared with healthy individuals

**Figure 2:**
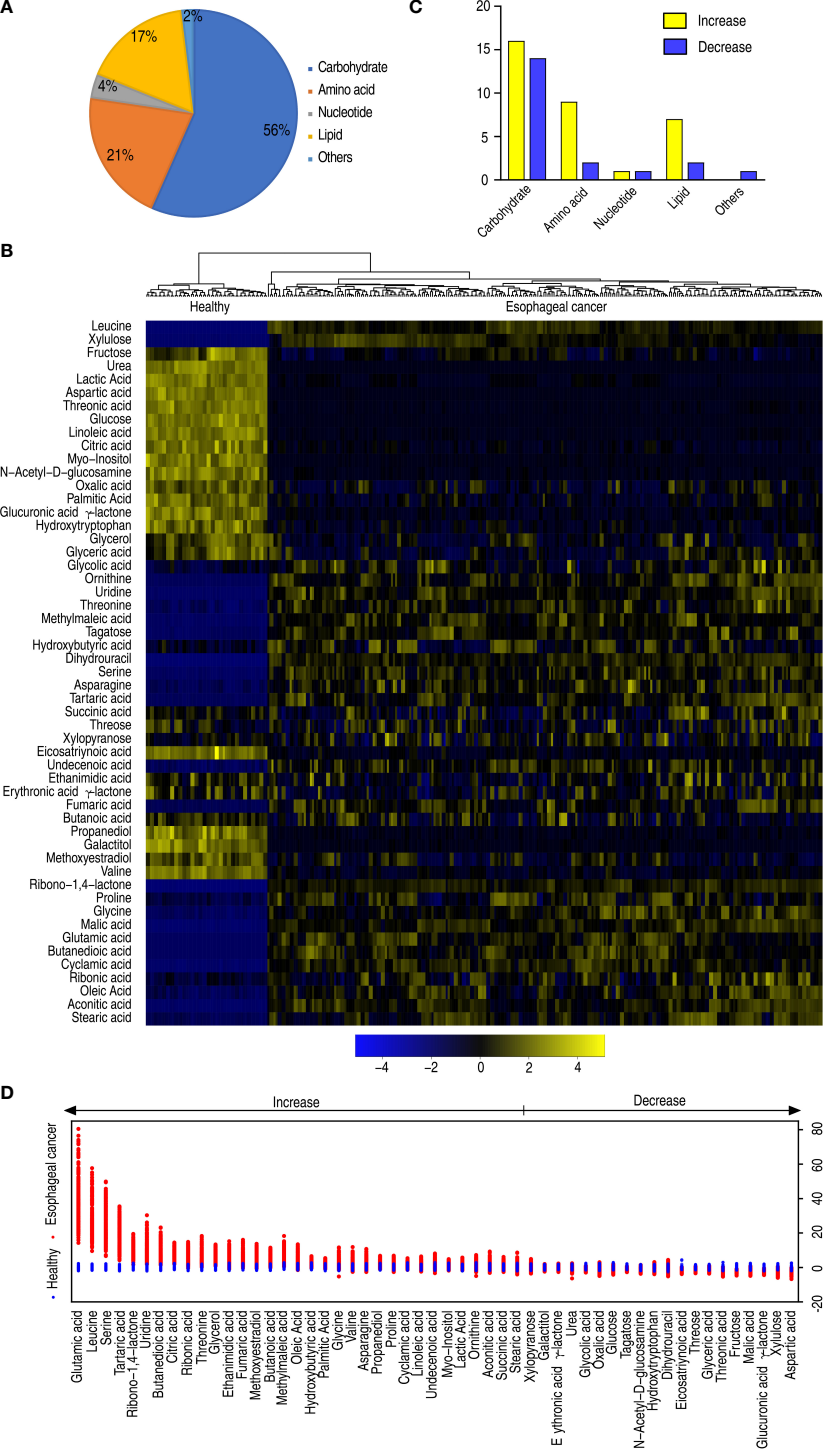
Differential metabolomes of patients with esophageal cancer. **(A)** Categories of the differential abundance of metabolites. **(B)** Heatmap showing differential abundance of metabolites. Yellow color and blue color indicate increase and decrease of metabolites relative to the median metabolite level, respectively (see color scale). **(C)** The number of differentially abundant metabolites is increased and decreased in every category. **(D)** *Z*-score plot of differential metabolites based on control (health). The data of the esophageal cancer group are scaled to the mean and standard deviation of control. Each point represents one metabolite in one technical repeat and is colored by sample types.

Among the 58 metabolites, 53 metabolites were differential in abundance, compared with healthy individuals (*p* < 0.05). These differential abundances of metabolites were categorized into carbohydrate (56%), amino acid (21%), nucleotide (4%), lipid (17%), and others (2%) (**Figure 2A**). Clustering analysis on these differential metabolites showed that healthy individuals and patients with esophageal cancer were separately clustered (**Figure 2B**), suggesting that there is an esophageal cancer-related metabolome. The number of upregulated and downregulated metabolites in these categories is shown in **Figure 2C**. Among these categories, amino acid and lipid were ranked as the first and second with the greatest difference in number between upregulated and downregulated metabolites, respectively (**Figure 2C**). *Z*-value analysis indicated how many standard deviations were away from the mean. The analysis showed more deviations in upregulated than downregulated metabolites. Glutamic acid, leucine, and serine were listed as the top three out of the upregulated metabolites (**Figure 2D**). Among the 53 differential metabolites, the abundance of 30 metabolites was positively or negatively correlated with disease stages (**Supplementary Figure 3**). These results suggest that the elevated amino acid metabolism is a characteristic feature in the esophageal cancer-related metabolome.

### Metabolic pathways of patients with esophageal cancer

**Figure 3:**
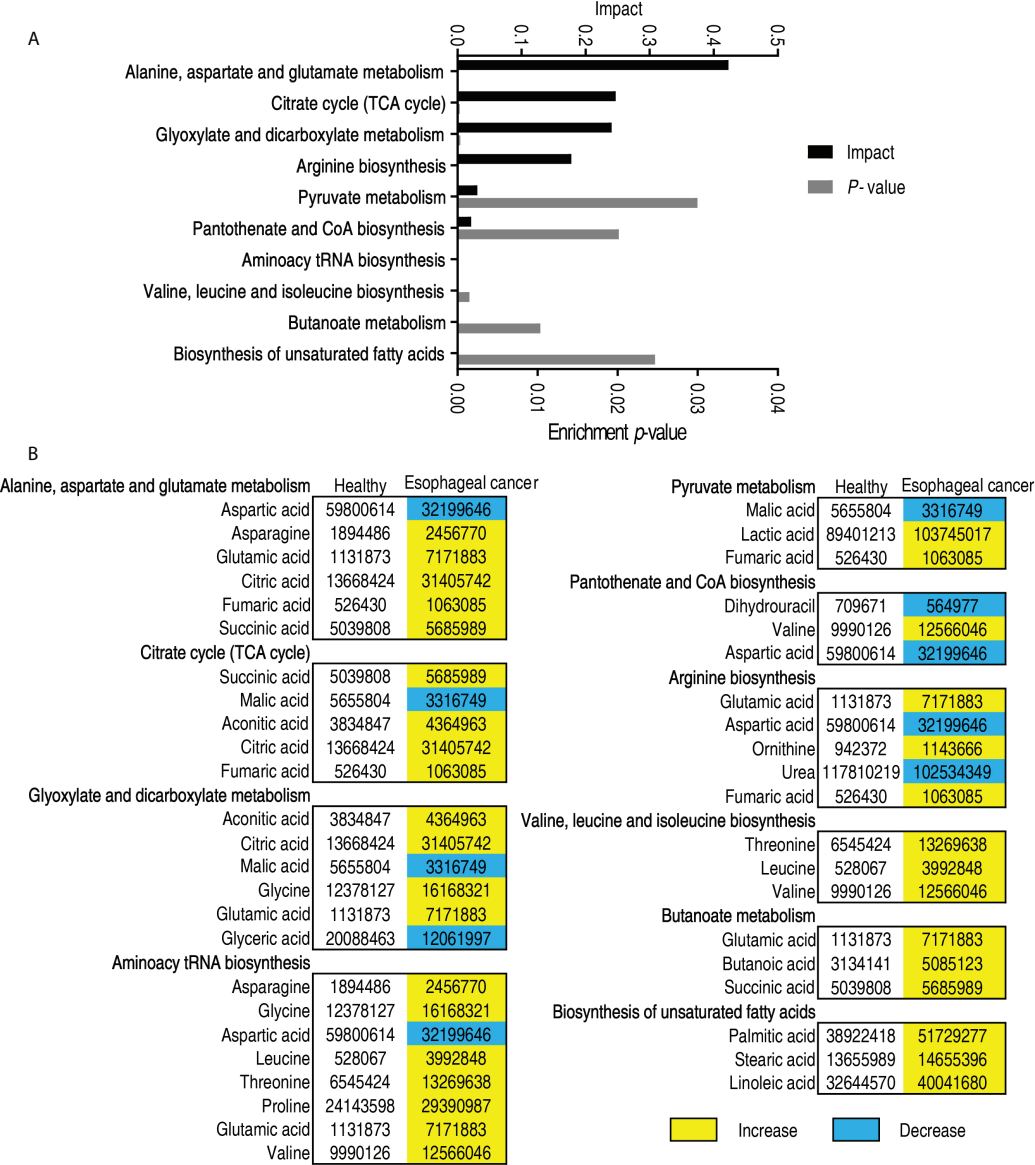
Pathway analysis of patients with esophageal cancer. **(A)** Pathway enrichment of differential abundant metabolites. **(B)** The volume of the differential metabolites of patients with esophageal cancer compared with normal individuals. Yellow and blue indicate increase and decrease of metabolites, respectively.

Metabolic pathways are a linked series of chemical reactions, leading to anabolism or breakdown of metabolites within a cell. Thus, investigation on differentially metabolic pathways is necessary to understand the difference between abnormal and normal metabolomes. To do this, 53 differentially abundant metabolites were analyzed using online software (http://www.metaboanalyst.ca). Ten metabolic pathways were enriched in patients with esophageal cancer. They were alanine, aspartate, and glutamate metabolism; citrate cycle (TCA cycle); glyoxylate and dicarboxylate metabolism; arginine biosynthesis; pyruvate metabolism; pantothenate and CoA biosynthesis; aminoacyl-tRNA biosynthesis; valine, leucine, and isoleucine biosynthesis; butanoate metabolism; and biosynthesis of unsaturated fatty acids (**Figure 3A**). Out of the 10 pathways, 5 (alanine, aspartate, and glutamate metabolism; glyoxylate and dicarboxylate metabolism; aminoacyl-tRNA biosynthesis; arginine biosynthesis; and butanoate metabolism) contained glutamic acid, whose difference was the greatest in the elevated differential metabolites; and 2 (aminoacyl-tRNA biosynthesis and valine, leucine, and isoleucine) included leucine, whose difference was the second in the elevated differential metabolites. On the other hand, among the 10 metabolic pathways, all metabolites were elevated in valine, leucine, and isoleucine biosynthesis; butanoate metabolism; and biosynthesis of unsaturated fatty acids, and most metabolites were increased in alanine, aspartate, and glutamate metabolism; TCA cycle; glyoxylate and dicarboxylate metabolism; aminoacyl-tRNA biosynthesis; arginine biosynthesis; and butanoate metabolism. In addition, five metabolites (aspartic acid, malic acid, glyceric acid, valine, and urea) were reduced in these enriched metabolic pathways (**Figure 3B**). These results indicate that the top five involved metabolic pathways include amino acid metabolism (alanine, aspartate, and glutamate metabolism, and arginine biosynthesis), the central carbon metabolism (TCA cycle and pyruvate cycle), and glyoxylate and discarboxylate metabolism.

### Biomarkers of patients with esophageal cancer

**Figure 4:**
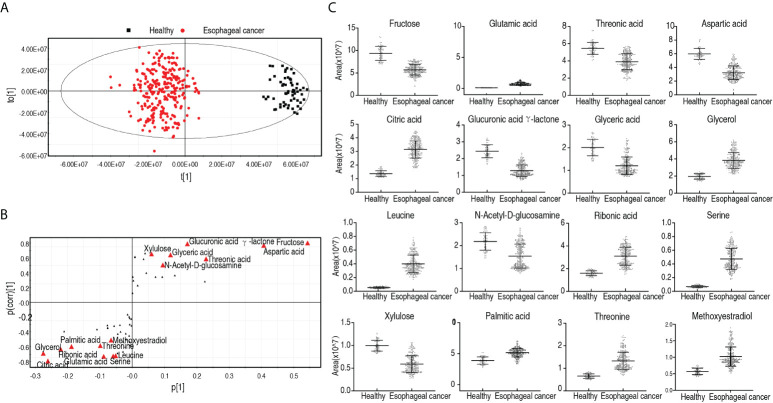
Identification for biomarkers in patients with esophageal cancer. **(A)** OPLS-DA analysis of healthy group and esophageal cancer group. Each dot represents one technical repeat in the plot. **(B)** S-plot is generated from OPLS-DA. The triangle represents metabolites and candidate biomarkers are highlighted in red. **(C)** Abundance of biomarkers. Results **(C)** are displayed as mean ± SEM.

In order to identify biomarkers that are most closely related to the carcinogenesis of esophageal cancer, OPLS-DA was used to perform this analysis. The score plot shows patients with esophageal cancer distinguished from healthy individuals, and correlation plots identified variables (**Figure 4A**). Discriminating variables were shown in the S-plot, where cutoff values were set as greater than or equal to 0.05 and 0.5 for the absolute value of *p*[1] and *p*(corr)[1], respectively. A total of 16 biomarkers were identified by component t[1], which differentiated most patients from healthy individuals and variants of the two groups. The 16 biomarkers included elevated glycerol, citric acid, ribonic acid, palmitic acid, methoxyestradiol, threonine, glutamic acid, serine, leucine and reduced fructose, aspartic acid, threonic acid, glucuronic acid γ-lactone, glyceric acid, and xylulose (**Figure 4B**). Among the nine elevated biomarkers, three (glutamic acid, aspartic acid, and serine), two (threonine and leucine), and one (palmitic acid) belong to non-essential amino acids, essential amino acids, and biosynthesis of fatty acids, respectively. Out of the six reduced biomarkers, four (fructose, threonic acid, glucuronic acid γ-lactone, and xylulose), one (aspartic acid), and one (glyceric acid) were classified into carbohydrate, amino acid, and lipid, respectively (**Figure 4C**). Out of them, elevation of glutamic acid, leucine, and serine was found at *p* < 0.05 in all esophageal cancer patients (**Supplementary Table 2**). In addition, metabolomes were compared between patients with early-stage esophageal cancer and healthy individuals as shown in **Supplementary Figures 4**–**6**. The resulting biomarkers were the same as those in the comparison between esophageal cancer patients and healthy individuals except for lactic acid, linoleic acid, and leucine, while lactic acid and linoleic acid were higher in early-stage esophageal cancer patients than healthy individuals (**Supplementary Figure 6**). These results suggest that the elevated amino acid metabolism is in sharp contrast to the reduced carbohydrate in patients with esophageal cancer.

### Metabolic features in patients with different malignant degrees of esophageal cancer

**Figure 5:**
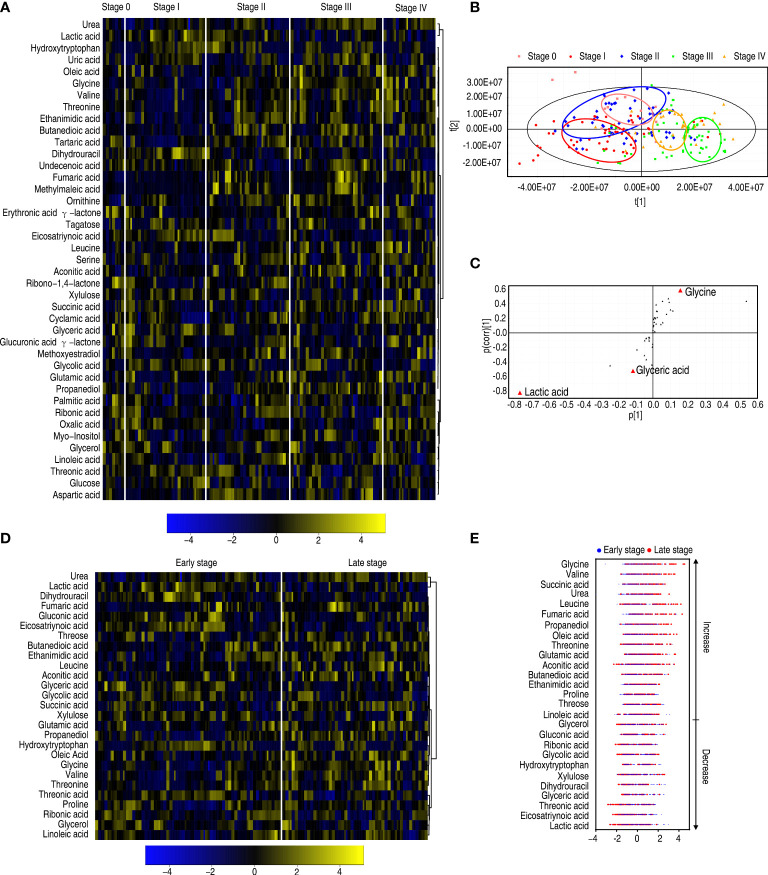
Differential metabolomes among stages 0, I, II, III, and IV of patients with esophageal cancer. **(A)** Heatmap showing five stages of esophageal cancer. **(B)** OPLS-DA of five stages of esophageal cancer. **(C)** S-plot is generated from OPLS-DA. The triangle represents metabolites and candidate biomarkers are highlighted in red. **(D)** Heatmap showing stage 0–II and stage III–IV esophageal cancer. **(E)** *Z*-score plot of differential metabolites based on control (stage 0–II esophageal cancer). The data of the stage III–IV esophageal cancer group were scaled to the mean and standard deviation of control. Each point represents one metabolite in one technical repeat and colored by sample types.

Furthermore, to explore whether the metabolic state is related to patients with different degrees of esophageal cancer, differential metabolic profiles were compared among patients with 0, I, II, III, and IV stages of esophageal cancer as shown in a heatmap (**Figure 5A**). OPLS-DA showed that most samples of each group were separately cycled but some overlapped between groups, suggesting that they possessed relatively unique metabolic characteristics. Specifically, stage 0 and stage I were separated but overlapped with stage II, while stages III and IV overlapped each other (**Figure 5B**). Component t[1] distinguished stages 0, I, and II (early stage) esophageal cancer from stages III and IV (late stage). Component t[2] separated variables of the five stages (**Figure 5B**). These results indicate that the metabolic state is related to stages of esophageal cancer. Among the five stages, the greatest difference in metabolites is detected between the early stage and the late stage. The S-plot showed that elevated glycine and urea, and reduced lactic acid and glyceric acid were biomarkers that identify the most significant difference (**Figure 5C**). Twenty-seven differential abundances of metabolites between the early and late stages were listed as a heatmap (**Figure 5D**). *Z*-scores were used to rank the differential abundance of metabolites in the late stage compared with the early stage. The top three increased metabolites were glycine, valine, and succinic acid (**Figure 5E**). They belong to amino acid and the TCA cycle, respectively. These results suggest that amino acid metabolism and the TCA cycle play a more significant role than others in the malignant degrees of esophageal cancer.

### Metabolic pathways in patients with different malignant degrees of esophageal cancer

**Figure 6:**
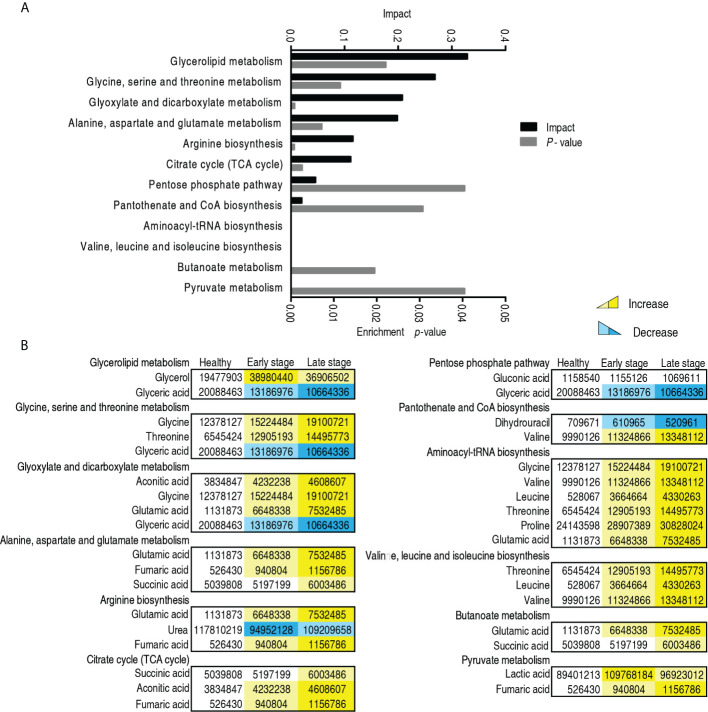
Pathway enrichment in the early and late -stages of patients with esophageal cancer. **(A)** Pathway enrichment of differential abundant metabolites. **(B)** The level of the differential metabolites of stage 0–II and stage III–IV esophageal cancer compared with healthy people. Yellow and blue indicate increase and decrease of metabolites, respectively.

To further understand the metabolic alterations between the early stage and the late stage in patients with esophageal cancer, MetaboAnalyst was used to identify altered pathways, leading to the enrichment of 11 metabolic pathways (**Figure 6A**). According to impact value, they were ranked as follows: glycerolipid metabolism; glycine, serine, and threonine metabolism; glyoxylate and dicarboxylate metabolism; alanine, aspartate, and glutamate metabolism; arginine biosynthesis; TCA cycle; pentose phosphate pathway; pantothenate and CoA biosynthesis; aminoacyl-tRNA biosynthesis; valine, leucine, and isoleucine biosynthesis; butanoate metabolism; and pyruvate metabolism. Among them, five pathways (glyoxylate and dicarboxylate metabolism; alanine, aspartate, and glutamate metabolism; arginine biosynthesis; aminoacyl-tRNA biosynthesis; and butanoate metabolism) were shared with those identified between patients with esophageal cancer and normal individuals described in **Figure 3**. The others were enriched specifically for malignant degrees of esophageal cancer, where glycerolipid metabolism and glycine, serine, and threonine metabolism were ranked as first and second, respectively. On the other hand, among the 11 enriched metabolic pathways, all metabolites were upregulated with disease stages in the TCA cycle; aminoacyl-tRNA biosynthesis; valine, leucine, and isoleucine biosynthesis; butanoate metabolism; and pyruvate metabolism, while metabolites of the other enriched metabolic pathways were upregulated or downregulated with disease stages except for pentose phosphate pathway (only glyceric acid was reduced with disease stages) (**Figure 6B**). These results indicate that the modulation of metabolic pathways is a characteristic feature of patients with esophageal cancer, which identified the early stage from the late stage. Glycine, the top increased metabolite, belongs to the second-ranked glycine, serine, and threonine metabolism, suggesting the importance of the amino acid and its metabolism in the malignant degrees of esophageal cancer.

### Biomarkers in patients with different malignant degrees of esophageal cancer

**Figure 7:**
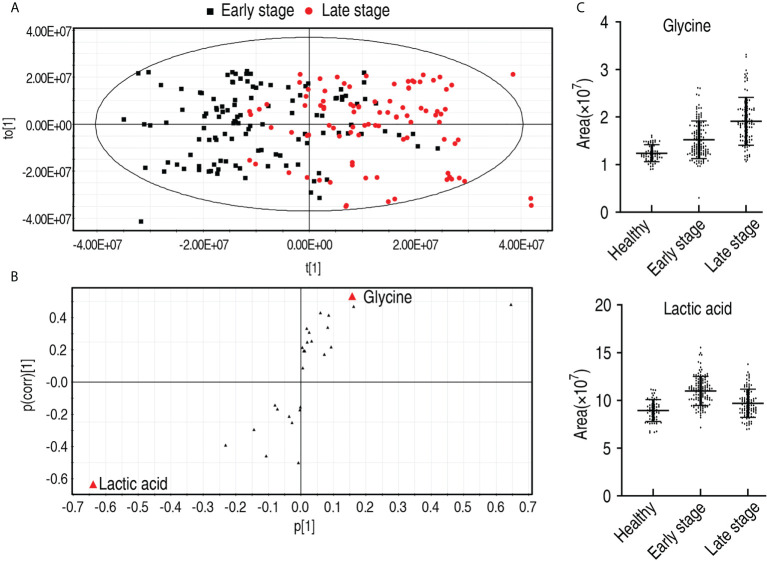
Identification for biomarkers in the early and late stages of patients with esophageal cancer. **(A)** OPLS-DA of stage 0–II and stage III–IV esophageal cancer. Each dot represents one technical repeat in the plot. **(B)** S-plot is generated from OPLS-DA. The triangle represents metabolites and candidate biomarkers are highlight in red. **(C)** Abundance of biomarkers. Results are displayed as mean ± SEM.

To find out which metabolites are related to the malignant degrees of esophageal cancer, OPLS-DA was conducted to recognize the sample pattern between the early stage and the late stage. Component t[1] mostly distinguished the early stage from the late stage, while component t[2] separated variables of the two stages (**Figure 7A**). Discriminating variables were shown by the S-plot, yielding two biomarkers by component t[1]. They were upregulated glycine and downregulated lactic acid (**Figure 7B**). However, compared with healthy individuals, only glycine was elevated with the healthy individuals, the early stage, and the late stage, while no difference in lactic acid was detected between healthy individuals and the late stage, which was lower than the early stage (**Figure 7C**). Glycine alone identified 70.1% early stage and late stage of esophageal cancer (**Supplementary Table 3**). These results indicate that the elevated glycine is related to the progression from the early stage to the late stage.

### Metabolic features of esophageal cancer patients who received treatment compared with patients who did not start treatment

**Figure 8:**
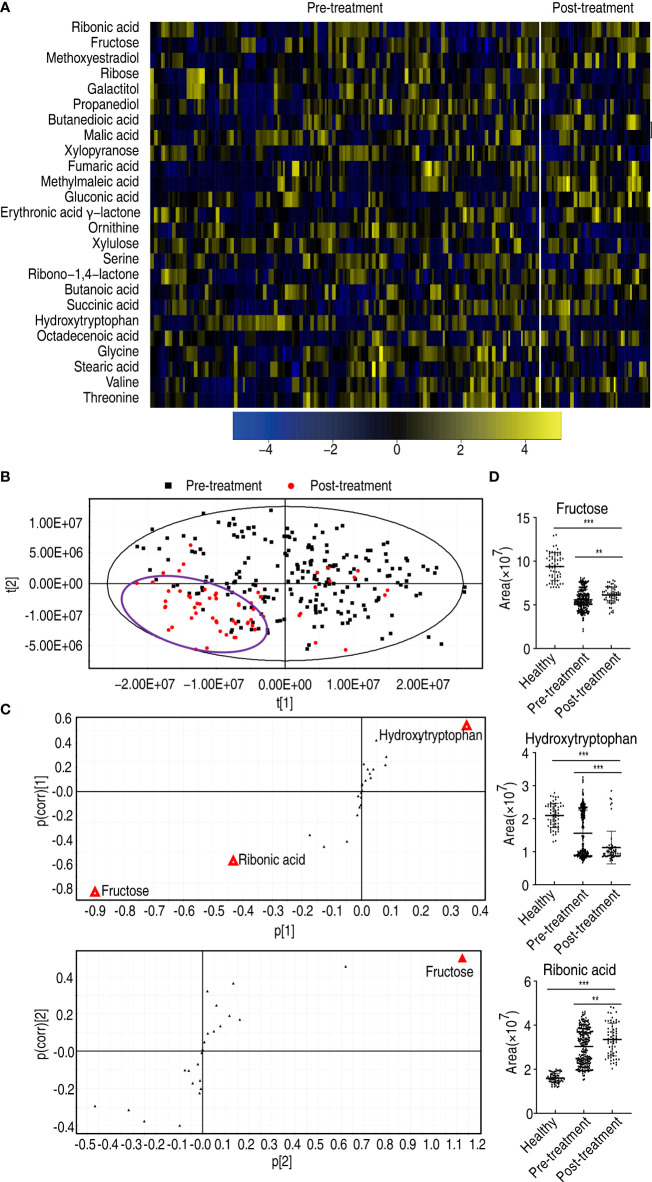
Differential metabolomes and biomarker identification between patients who received treatment and patients who did not receive treatment. **(A)** Heatmap showing differential metabolites. **(B)** PLS-DA of esophageal cancer before and after treatment. **(C)** S-plot is generated from PLS-DA. The triangle represents metabolites and candidate biomarkers are highlighted in red. **(D)** Abundance of biomarkers. Results are displayed as mean ± SEM, **p < 0.01, and ***p < 0.001 as determined by Student’s *t*-test.

Moreover, to explore whether there were metabolic biomarkers related to the prognosis, comparative metabolomics was performed in patients who received treatment compared to patients who did not receive treatment. Twenty-five differential abundances of metabolites were determined (**Figure 8A**). Then, PLS-DA was carried out to recognize the sample patterns of metabolomes as previously described (17) (here, PLS-DA is more sensitive than OPLS-DA). Most post-treatment samples were located in the left lower quadrant; the others and pre-treatment samples were distributed in the other three quadrants (**Figure 8B**). The variables responsible for components t[1] and t[2] were fructose, ribonic acid, hydroxytryptophan, and fructose (**Figure 8C**). Fructose overlapped between components t[1] and t[2], suggesting that fructose is a potential biomarker to differentiate the patients who received treatment from the patients who did not receive treatment. Compared with the healthy individuals, low fructose was detected in patients with esophageal cancer, but fructose was higher in the patients who received treatment than those who did not (**Figure 8D**). There were no difference in fructose abundance between male and female, and between those aged ≤60 and those aged >60 (**Supplementary Figure 7**). However, the abundance of hydroxytryptophan and ribonic acid was lower and higher, respectively, in the patients who received treatment than those who did not, followed by healthy individuals. Thus, only fructose may be selected as a potential biomarker to predict the treatment efficacy.

### Key metabolome changed with treatment in esophageal cancer patients

**Figure 9:**
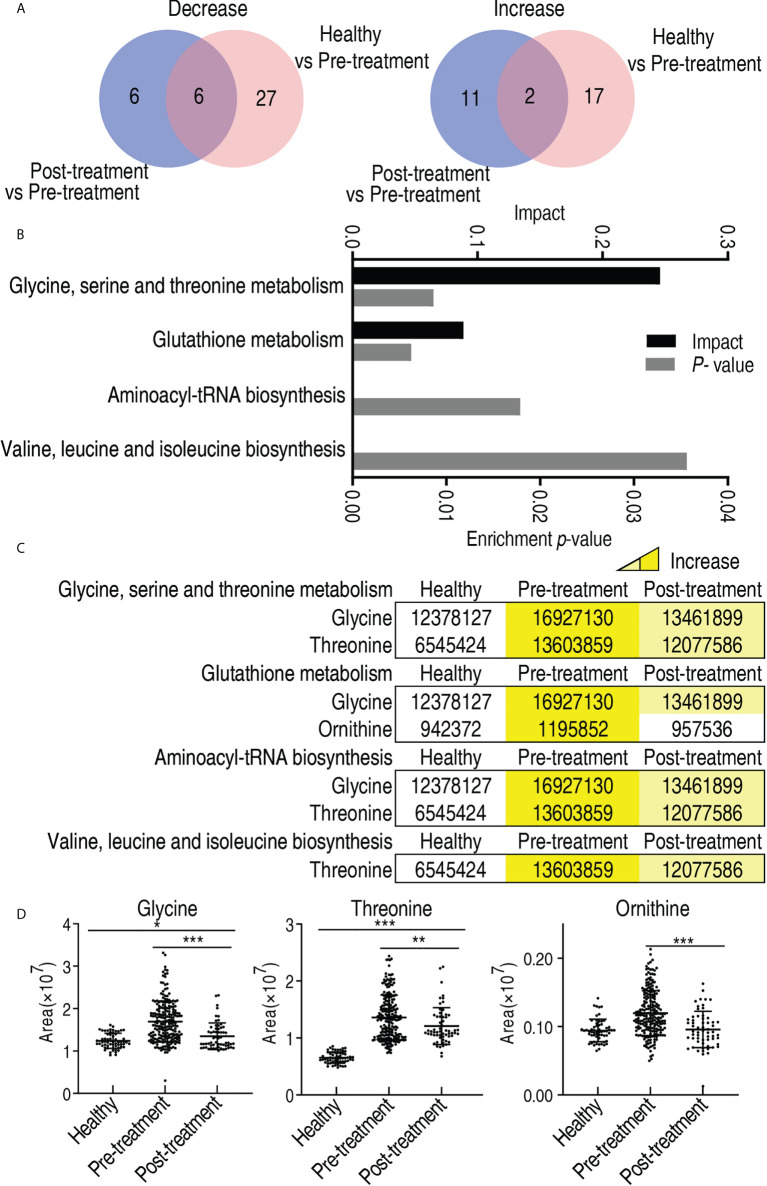
Differential metabolites and metabolic pathway enrichment between patients who received treatment and patients who did not receive treatment. **(A)** Venn diagram for overlapped and unique metabolites between healthy individuals/patients who did not receive treatment (pre-treatment) and patients who received treatment/patients who did not receive treatment (pre-treatment). **(B)** Pathway enrichment of differential abundant metabolites. **(C)** Abundance of the differential metabolites of pre-treated and post-treated esophageal cancer patients compared with healthy individuals. Yellow and blue indicate increase and decrease of metabolites, respectively. **(D)** Abundance of biomarkers. Results are displayed as mean ± SEM. *p < 0.05, **p < 0.01, and ***p < 0.001 as determined by Student’s *t*-test.

To further explore the biomarkers that potentially predicted treatment efficacy, comparative metabolomics was carried out between healthy individuals and the patients who did not receive treatment, and between the patients who received treatment and the patients who did not. The comparison between healthy individuals and the patients who did not receive treatment identified 33 decreased and 19 increased differential metabolites, while the comparison between the patients who received treatment and the patients who did not determined 12 decreased and 13 increased differential metabolites (*p* < 0.05). Among them, six decreased (glycine, propanediol, threonine, ornithine, stearic acid, and serine) and two increased metabolites (erythronic acid γ-lactone and fructose) overlapped in the two comparisons (**Figure 9A**). Then, pathway enrichment analysis was performed on the eight overlapped metabolites. Among them, three metabolites (glycine, threonine, and ornithine) worked, leading to enrichment of four metabolic pathways, namely, glycine, serine, and threonine metabolism; glutathione metabolism; aminoacyl-tRNA biosynthesis; and valine, leucine, and isoleucine biosynthesis (**Figure 9B**). The abundance of the three metabolites ranked from lowest to highest as follows: healthy individuals, the patients who received treatment, and the patients who did not receive treatment (**Figure 9C**). There was a significant difference among the three groups for glycine and threonine, but no difference was found between healthy individuals and the patients who received treatment for ornithine (**Figure 9D**). Notably, no difference in abundance of glycine, threonine, and ornithine was detected between male and female, and between those aged ≤60 and those aged >60 (**Supplementary Figure 7**). These results indicate that glycine, threonine, and ornithine may be selected as potential biomarkers to predict the treatment effect.

### Evaluation of glycine, threonine, ornithine, and fructose as an array for laboratory biomarkers

**Figure 10:**
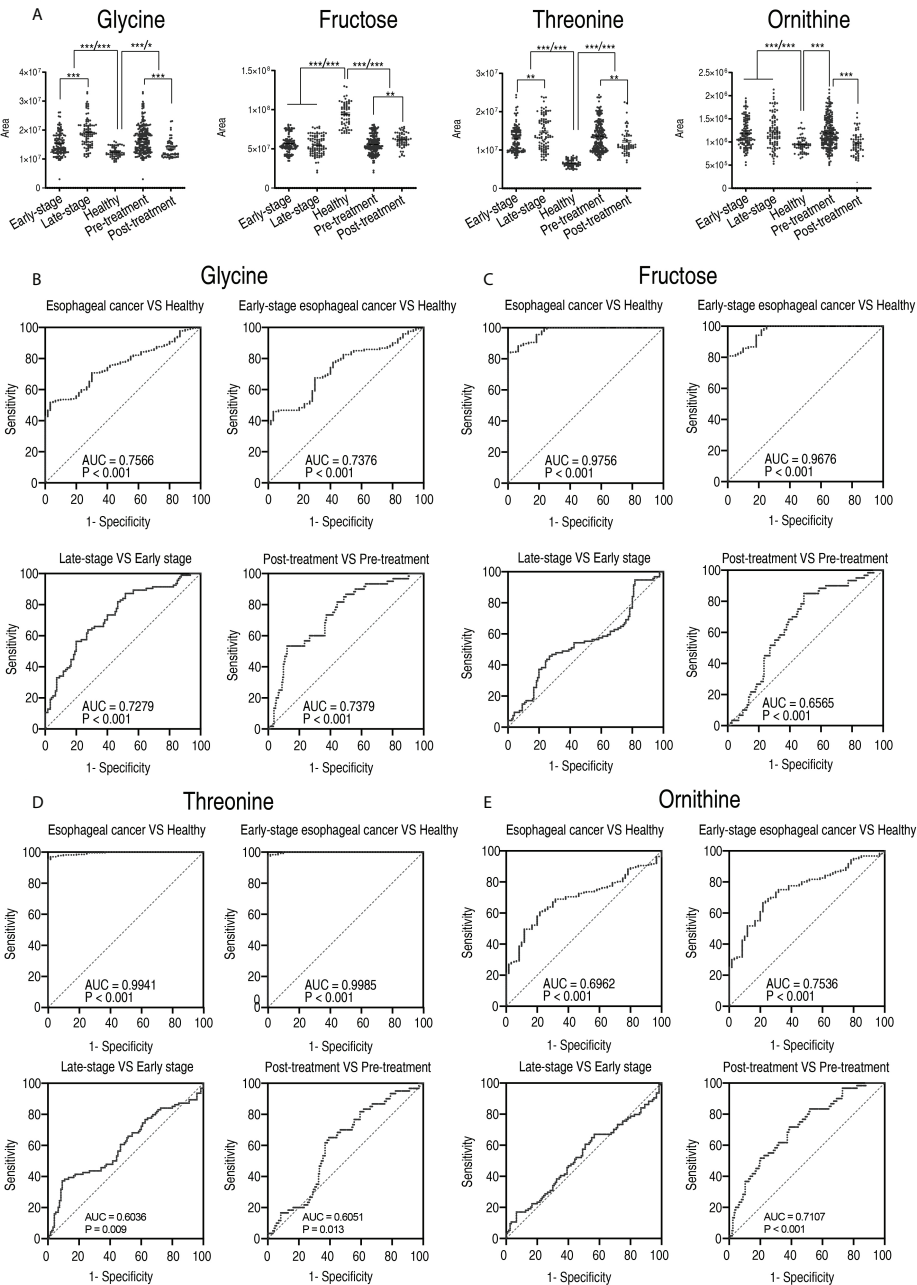
The clinical potential of glycine. **(A)** Comparison of glycine, fructose, threonine, and ornithine between healthy subjects and patients with early stage and late stage of esophageal cancer, and between healthy subjects, patients who received treatment (post-treatment), and patients who did not receive treatment (pre-treatment). Results are displayed as mean ± SEM, *p < 0.05, **p < 0.01, and ***p < 0.001 as determined by Student’s *t*-test. **(B–D)** ROC curves to distinguish esophageal cancer patients from normal individuals, early-stage esophageal cancer patients from healthy subjects, late-stage esophageal cancer patients from early-stage esophageal cancer patients, and post-treatment patients from pre-treatment by glycine **(B)**, fructose **(C)**, threonine **(D)**, and ornithine **(E)**, respectively.

The abundance of glycine, threonine, and ornithine elevated with disease progression from the early stage to the late stage and was lower in patients who received treatment than those who did not. In contrast, the abundance of fructose decreased with the disease progression and was higher in patients who received treatment than those who did not (**Figure 10A**). Further analysis showed that the receiver operating characteristic (ROC) curve of the four potential biomarkers yielded an area under the curve (AUC) from 0.6036 to 0.9985 (*p*-value = 0.013–<0.001) except for comparison between the early stage and the late stage in ornithine and fructose, where no difference was found (**Figures 10B–E**). Glycine, ornithine, threonine, and fructose identified 80.3%, 60.6%, 58.4, and 58.4% of the patients who received treatment from the patients who did not receive treatment, respectively. The combination of glycine with the other three differentiated 95.6% of the patients tested (**Supplementary Table 4**). These results suggest that an array for the four biomarkers can be regarded as a potential esophageal cancer serum biomarker for therapeutic effect.

## Discussion

Esophageal cancer is the most invasive disease associated with inclusive poor prognosis worldwide, with a dramatic increase in incidence in the Western world occurring over the past few decades (18). The poor prognosis and rising incidence of esophageal cancer highlight the need for improved prediction methods that are essential to disease progression and therapeutic efficacy (19). Here, a GC-MS-based metabolomics approach is used to understand the pathogenesis of esophageal cancer and identify diagnosis biomarkers to improve the diagnosis, prognosis, and treatment efficacy of patients with esophageal cancer. Glutamic acid, leucine, and serine are identified as the top three upregulated metabolites. Amino acid metabolism, fatty acid biosynthesis, the TCA cycle and pyruvate metabolism of the central carbon metabolism, and glyoxylate and discarboxylate metabolism are enriched as characteristic features in the metabolic profile of patients with esophageal cancer. Further analysis indicates that the greatest metabolic difference is determined between the early stage (0–II) and the late stage (III and IV). Glycerolipid metabolism; glycine, serine, and threonine metabolism; and glyoxylate and dicarboxylate metabolism as well as the reduced glycerate and the elevated glycine within these pathways play a more important role than other metabolic pathways and metabolites. Finally, the present study reveals that the reduced fructose and elevated glycine, threonine, and ornithine are the potential indicators that evaluate the efficacy of the therapy. Further studies are needed to verify them, but these findings indicate that the patients with esophageal cancer have a characteristic metabolic shift, which is changed with disease progression and therapeutic effect. Therefore, patients with esophageal cancer have metabolomes that are different from those of healthy individuals, which can be designed as esophageal cancer metabolome as described in other areas, such as antibiotic-resistant metabolome and anti-infective metabolome (20–23).

A line of evidence has shown that characteristic alterations have been determined in esophageal cancer metabolome including samples from serum, tumor and stroma tissue, and urine (11, 24, 25). However, among the studies with serum samples, no investigations have more than 80 specimens. The present study characterizes the changes in esophageal cancer metabolome in approximately two- to fivefold as many plasma specimens as the previous reports. Ten metabolic pathways are enriched and 15 biomarkers are identified. Among them, glycerolipid metabolism and alanine, aspartate, and glutamate metabolism as the top two affected metabolic pathways and glutamic acid as the most elevated metabolite are not revealed in these reports. These previously unknown metabolic pathways and biomarkers will be helpful in further understanding the pathogenesis of esophageal cancer. Importantly, the present study identifies the elevated amino acid metabolism in sharp contrast to the reduced carbohydrate as a consequence of esophageal cancer.

A core finding is that the metabolomes of early stage (0–II) and those of late stage (III–IV) are different, indicating that the differential metabolome may be used to judge disease progression. Buck et al. suggest that the metabolic constitution of tumor is superior to tumor regression grading for evaluating response to neoadjuvant therapy of patients with EAC. They identify GDP-glucose, dADP, nicotinate nucleotide, and acetyl-D-glucosamine as the biomarkers (26). Zhang et al. investigate the serum metabolomes of 25 patients with esophageal cancer and identify significant changes in lipid metabolism, amino acid metabolism, glycolysis, ketogenesis, tricarboxylic acid (TCA) cycle, and energy metabolism. These results demonstrate that metabolic profiling of serum could be useful as a screening tool for early EC diagnosis and prognosis (27). The present study highlights the crucial role of the previously unknown glycine, serine, and threonine metabolism and glycerolipid metabolism as well as the elevated glycine in the malignant degrees of esophageal cancer.

Another core finding is that the metabolic profiling of plasma could be useful as a screening tool for therapeutic effect in patients with esophageal cancer. The biomarkers between the patients who received treatment and the patients who did not receive treatment revealed features resembling the fructose, glycine, threonine, and ornithine concept. Specifically, the elevation of fructose and the decrease of glycine, threonine, and ornithine are related to the therapeutic efficacy. Liu et al. compare serum metabolomes between pre- and post-esophagectomy in 34 treatment-naive patients with ESCC and reveal 12 ESCC tumor-associated serum metabolites with potential for monitoring therapeutic efficacy and disease relapse. They are phenylalanine hydroxyproline, pipercoic acid, maltose, decanoic acid, nonanoic acid, 1,5-dehydrated glucoside, hydroxybenzoic acid, glycolic acid, 2-pyrrolidone oleic acid, and glyceryl phosphate. These authors highlight serum pipecolic acid as an attractive biomarker for predicting ESCC tumorigenesis (28). The present study stressed on the importance of the elevated fructose and the reduced glycine, threonine, and ornithine in treatment efficacy, which is consistent with the above conclusion that the elevated amino acid metabolism is in sharp contrast to the reduced carbohydrate as a characteristic feature of esophageal cancer.

Finally, comparison between esophageal cancer patients and healthy individuals identifies elevated glycerol, citric acid, ribonic acid, palmitic acid, linoleic acid, threonine, glutamic acid, serine, and leucine, and reduced fructose, aspartic acid, threonic acid, glucuronic acid γ-lactone, glyceric acid, and xylulose as biomarkers. Among the 15 biomarkers, glutamic acid, leucine, and serine differentiate all patients from the control, while citric acid, aspartic acid, ribonic acid, threonine, glycerol, glucuronic acid γ-lactone, xylulose, and fructose identify 87.4%–97.6% of the patients. The combination of glutamic acid, leucine, and serine, probably with others, will provide an array to identify patients with esophageal cancer from healthy individuals. Out of the 15 biomarkers identified in the present study, glutamic acid, serine, leucine, linoleic acid, and citric acid were reported in previous literature (27, 29–32). Therefore, the present study provides new biomarkers for auxiliary identification of esophageal cancer.

Importantly, these findings can be used not only as a clue to explore metabolic mechanisms underlying esophageal cancer progression, but also as potential candidates to develop laboratory biomarkers for diagnosis and prediction of clinical outcomes in esophageal cancer. Among the identified potential metabolic pathways and biomarkers, glycine, serine, and threonine metabolism and glycine are first recommended. Glycine is the only biomarker and the one with the most potential to predict the disease progress and the treatment effect, respectively. Glycine, serine, and threonine metabolism is the second ranked metabolic pathway in terms of impact in the late stage compared with those in the early stage and glycine. Glycine belongs to glycine, serine, and threonine metabolism. The overlapping suggests the importance of the pathway in the pathogenesis of esophageal cancer and of the metabolite as a potential laboratory biomarker. Then, glycine, ornithine, threonine, and fructose are recommended as a potential array to evaluate treatment efficacy. Correspondingly, the elevated amino acid metabolism is in sharp contrast to the reduced carbohydrate, which provides a clue for the mechanistic understanding of esophageal cancer. Finally, glutamic acid, leucine, and serine are recommended as a potential array and an auxiliary indicator in the diagnosis of esophageal cancer.

In summary, the present study uses GC-MS-based metabolomes to understand the metabolic shift of esophageal cancer. The elevated amino acid metabolism in sharp contrast to the reduced carbohydrate is defined as a characteristic feature of the disease. The metabolic shift is related to the difference between esophageal cancer patients and healthy individuals, the progression from the early stage to the late stage, and the treatment efficacy. Specifically, there are three key findings: (1) Elevation of glutamic acid, leucine, and serine is a marker to identify esophageal cancer patients from healthy individuals. (2) Glycine level is positively related to disease progression. (3) The combination of glycine with fructose, threonine, and ornithine provides an array to identify patients who received treatment from those who did not. These findings not only provide biomarkers for laboratory diagnosis, but also offer an important clue to understand the metabolic mechanisms of esophageal cancer pathogenesis. However, the patients in the early-stage and late-stage groups and the pre- and post-treatment groups were not the same. Therefore, these identified metabolites as well as pathways can be considered potential pathways and biomarkers, but further studies are needed to verify them. Furthermore, the subjects used are not matched in terms of number, age, sex, and other interventions, thereby confounding non-matched variables that may influence the metabolomics outcomes. In addition, there is no information on obesity and there is limited information on smoking habit in the study.

## Materials and methods

### Clinical sample collection and pretreatment

We collected a total of 167 serum samples from all patients with esophageal cancer from the First Affiliated Hospital of Zhengzhou University and Henan Cancer Hospital from November 2017 to August 2020, and collected a total of 30 serum samples from patients who came to the hospital for medical examinations during the same period. When all samples were collected, the individuals were in a fasting state and were not injected with nutrients such as glucose, amino acids, and fat emulsions, and the skin was routinely sterilized according to clinical practice. Two milliliters of peripheral venous whole blood was drawn, and immediately centrifuged at 4,000 rpm for 5 min to obtain serum.

### Metabolomic profiling

Metabolomic profiling was performed as previously described (33, 34). An aliquot of 1 ml of −80°C pre-cooling methanol (Sigma) was added to 100 μl of serum and the mixture was vortexed for 1 min. Then, at 4C, the mixture was centrifuged for 10 min at a rotation speed of 12,000 rpm. After that, 1 ml of supernatant was transferred to a 1.5-ml centrifuge tube. To normalize variations across all samples, an internal standard (0.1 mg/ml ribitol) (Sigma) was added to the supernatant and dried in a vacuum centrifuge. Then, 80 μl of 20 mg/ml methoximation-pyridine hydrochloride was added to the dried samples, dissolving ultrasonically and reacting for 3 h at 37°C, followed by adding 80 μl of N-methyl-N-(trimethylsilyl) trifluoroacetamide (MSTFA, Sigma) for reaction for 30 min at 37°C. The derivatized sample with 1 μl was injected to a DBS-MS column. The initial temperature was 85°C for 5 min, followed by an increase to 270°C at a rate of 15°C/min and held for 5 min. Helium was used as the carrier gas at a constant flow rate of 1 ml/min. The MS scan range was at 50–600 m/z. GC-MS data were detected with an Agilent 7890A GC equipped with an Agilent 5975C VL MSD detector (Agilent Technologies). Two technical repeats were prepared for each sample. QC was performed as described previously (35–37). Pooled QC samples were collected by equally mixing small aliquots (10 μl) of sera from the samples studied, and it was prepared in the same way as the samples described above for testing. One QC sample was run after every 14 samples.

### Metabolic profiling analysis

Data analysis was carried out as previously described (33, 34). Agilent software (Agilent 6.0) was used for initial peak detection and mass spectrometry deconvolution, and the National Institute of Standards and Technology (NIST) Mass Spectral Library was employed for metabolite identification, and then data matrix standardization was done using an internal standard (ribitol) and the total intensity; finally, hierarchical clustering was performed on the R platform (R × 64 4.0.3). The software IBM SPSS Statistics 22 was used to conduct a significant difference analysis (non-parametric test) on the standardized data, and metabolites with a *p*-value of less than 0.05 were considered significant. PLS-DA and OPLS-DA were carried out on SIMCA-P (version 12; Umetrics, Umea, Sweden) (38). *Z*-score was used to analyze the degree of dispersion of different metabolites after normalized area. MetaboAnalyst 5.0 (http://www.metaboanalyst.ca) was used to enrich the pathways of differential metabolites, and metabolic pathways with a *p* value <0.05 were drawn (39). GraphPad Prism 9.0 was used to draw figures. ROC analysis was performed using IBM SPSS Statistics 22 to obtain the AUC value and Youden index, and the cutoff value was obtained according to Youden index. Spearman analysis was carried out on IBM SPSS Statistics 22 to yield correlation coefficients between metabolites and esophageal cancer progression.

## Data availability statement

The raw data supporting the conclusions of this article will be made available by the authors, without undue reservation.

## Ethics statement

This study was reviewed and approved by The ethics review committee of Zhengzhou University. The patients/participants provided their written informed consent to participate in this study. Written informed consent was obtained from the individual(s) for the publication of any potentially identifiable images or data included in this article.

## Author contributions

JZ wrote the manuscript. JZ, HL and XP conceptualized and designed the project. HL and XP interpreted the data. PW and HY collected samples. MJ and YS processed the samples for GC/MS loading. XY, MJ, YS, PW and HY performed data analysis. All authors contributed to the article and approved the submitted version.

## Funding

This work was supported by grants from the State Key Laboratory of Esophageal Cancer Prevention and Treatment (2020 for Ming Jiang and 2021 for Yu-bin Su), the Science and Technology Project of Guangzhou (201904020042), and the NSFC project (31772888).

## Conflict of interest

The authors declare that the research was conducted in the absence of any commercial or financial relationships that could be construed as a potential conflict of interest.

## Publisher’s note

All claims expressed in this article are solely those of the authors and do not necessarily represent those of their affiliated organizations, or those of the publisher, the editors and the reviewers. Any product that may be evaluated in this article, or claim that may be made by its manufacturer, is not guaranteed or endorsed by the publisher.

## Supplementary material

Supplementary Figure 1 Quality control of GC-MS based metabolomics.

Supplementary Figure 2 OPLS-DA analysis of global metabolites in esophageal cancer *vs* healthy.

Supplementary Figure 3 Correlation analysis between differential metabolites and progression of esophageal cancer.

Supplementary Figure 4 Differential metabolomes of early-stage esophageal cancer patients. A. Categories of the differential abundance of metabolites. B. Heatmap showing differential abundance of metabolites. Yellow color and blue color indicate increase and decrease of metabolites relative to the median metabolite level, respectively (see color scale). C. Number of differentially abundant metabolites is increased and decreased in every category. D. Z-score plot of differential metabolites based on control (healthy).

Supplementary Figure 5 Pathway analysis of early-stage esophageal cancer patients. A. Pathway enrichment of differential abundant metabolites. B. The levels of the differential metabolites.

Supplementary Figure 6 Identification for biomarkers in patients with early-stage esophageal cancer. A. OPLS-DA of healthy group and early-stage esophageal cancer group. B. S-plot is generated from OPLS-DA. C. Abundance of biomarkers. Results (C) are displayed as mean ± SEM.

Supplementary Figure 7 Levels of metabolites in esophageal cancer patients with different gender and age. A. Levels of fructose, glycine, ornithine and threonine in patients with esophageal cancer of different genders. B. Abundance of fructose, glycine, ornithine and threonine in patients with esophageal cancer at different age groups (≤ 60 or > 60).
