# Supplementary figures and images for: Untargeted serum metabolomics reveals potential biomarkers and metabolic pathways associated with esophageal cancer
Source: Front Oncol. 2022 Sep 13;12:938234. doi: 10.3389/fonc.2022.938234 (PMC9513043; doi:10.3389/fonc.2022.938234)

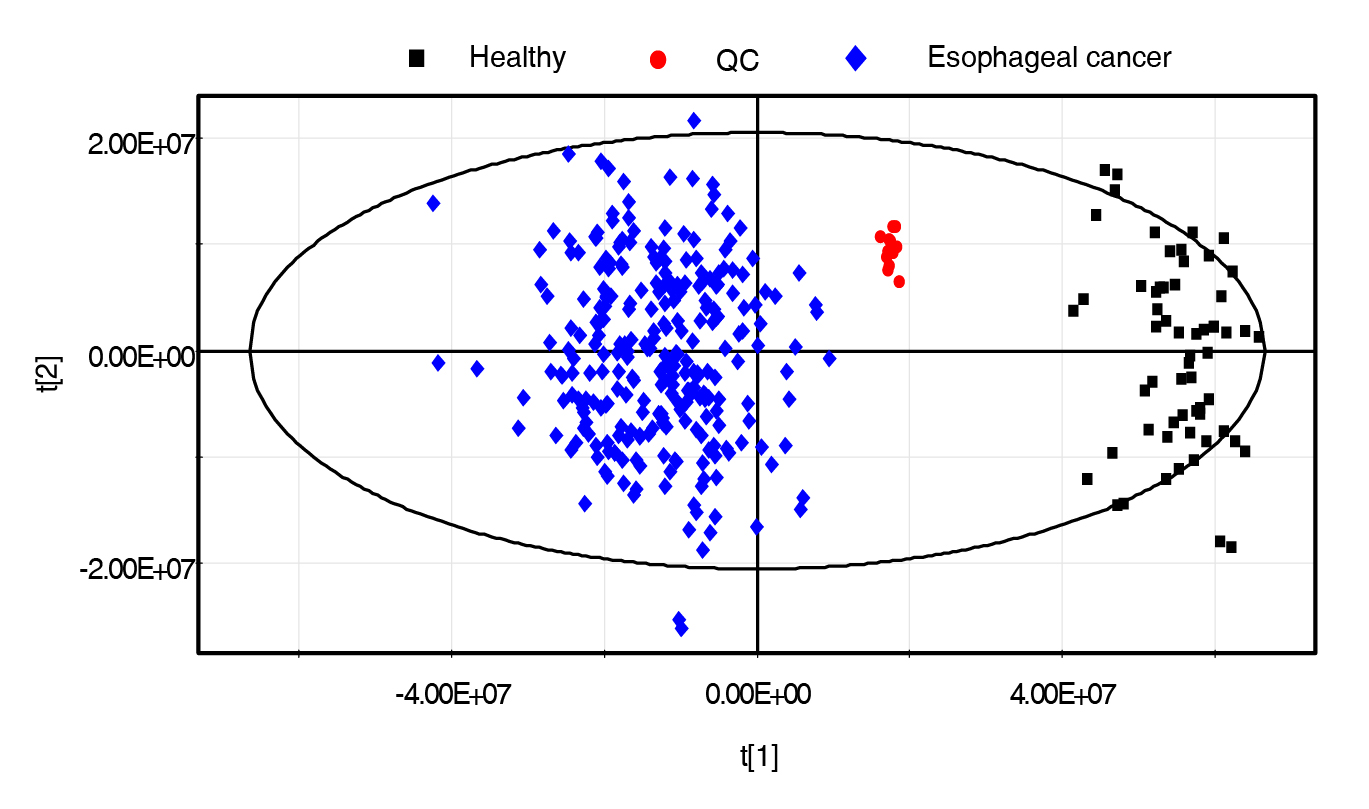

Supplement: Supplementary Figure 1 — Quality control of GC-MS based metabolomics. [file Image_1.jpeg]

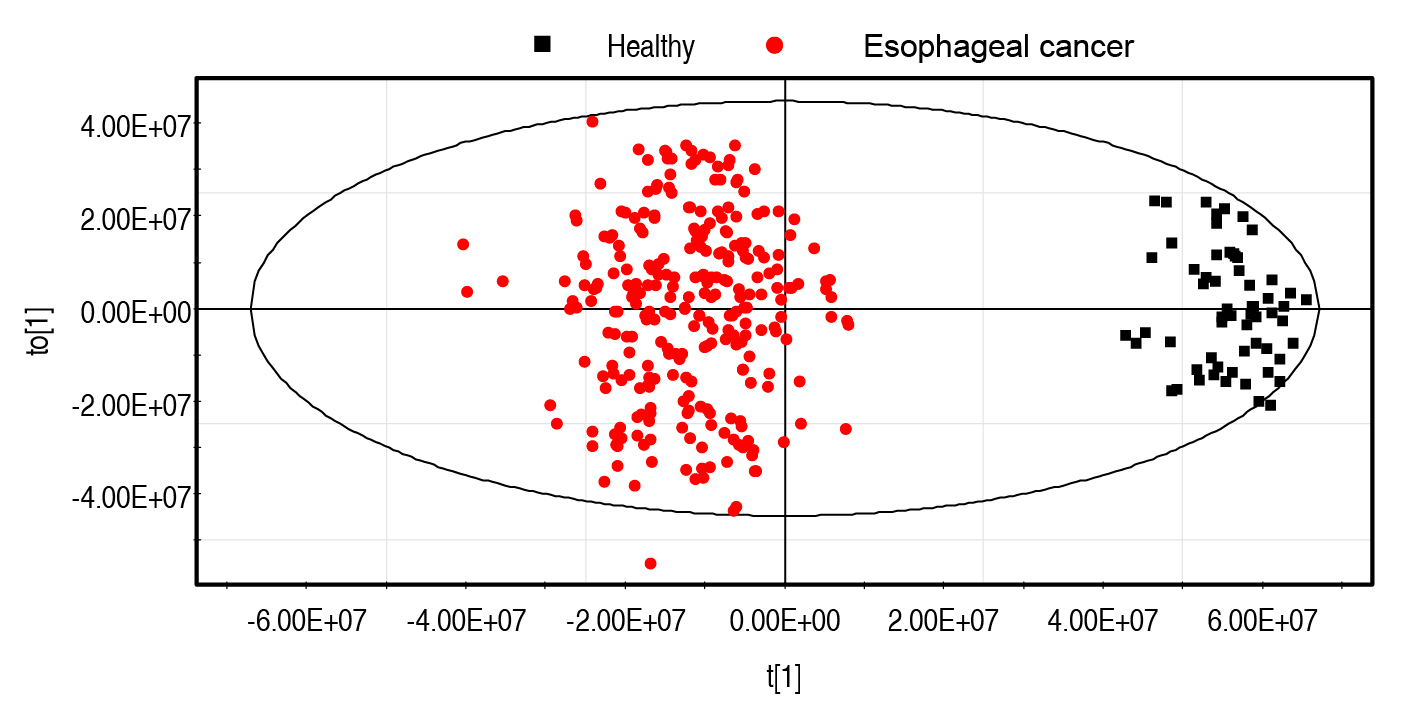

Supplement: Supplementary Figure 2 — OPLS-DA analysis of global metabolites in esophageal cancer vs healthy. [file Image_2.jpeg]

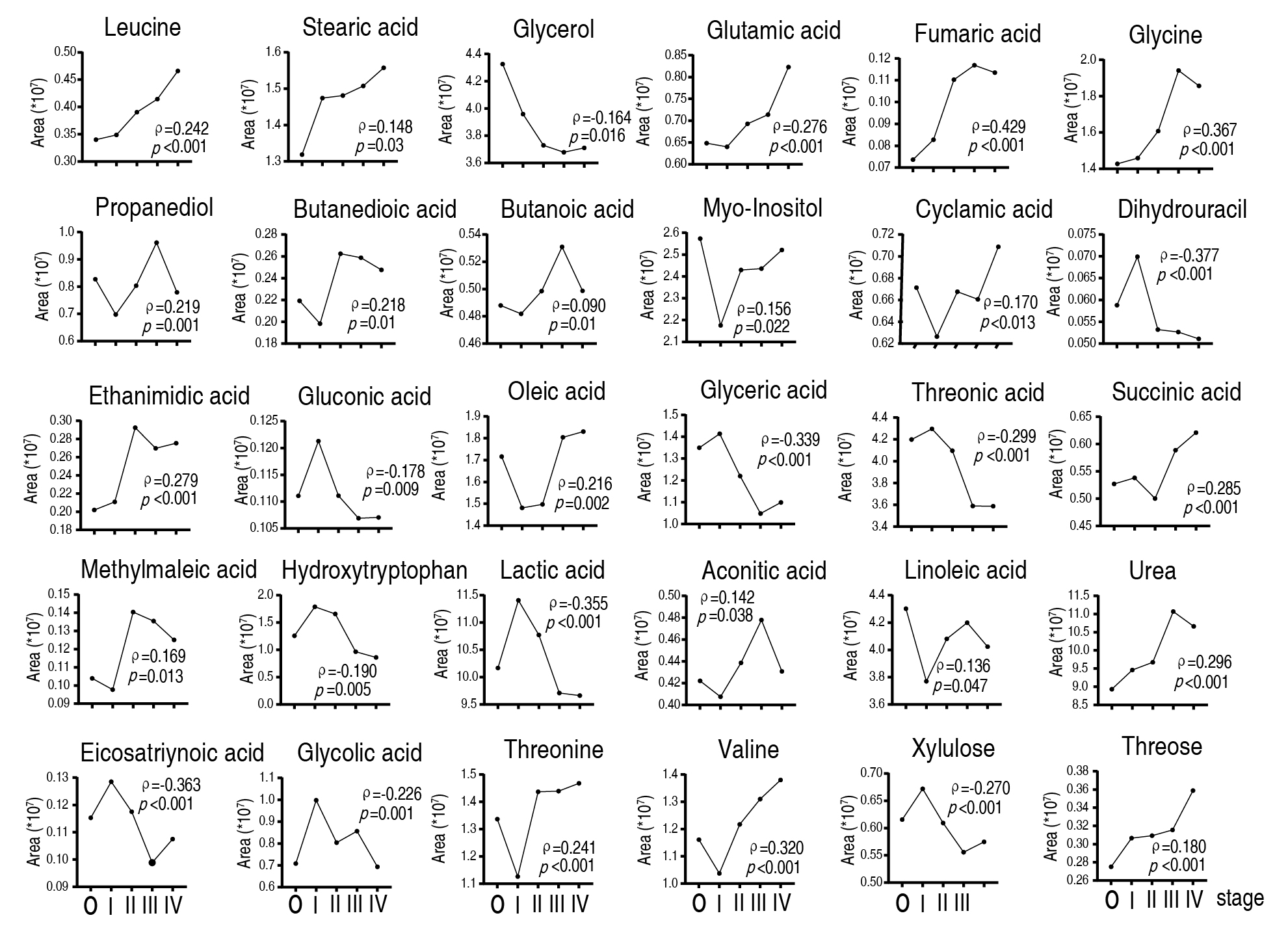

Supplement: Supplementary Figure 3 — Correlation analysis between differential metabolites and progression of esophageal cancer. [file Image_3.jpeg]

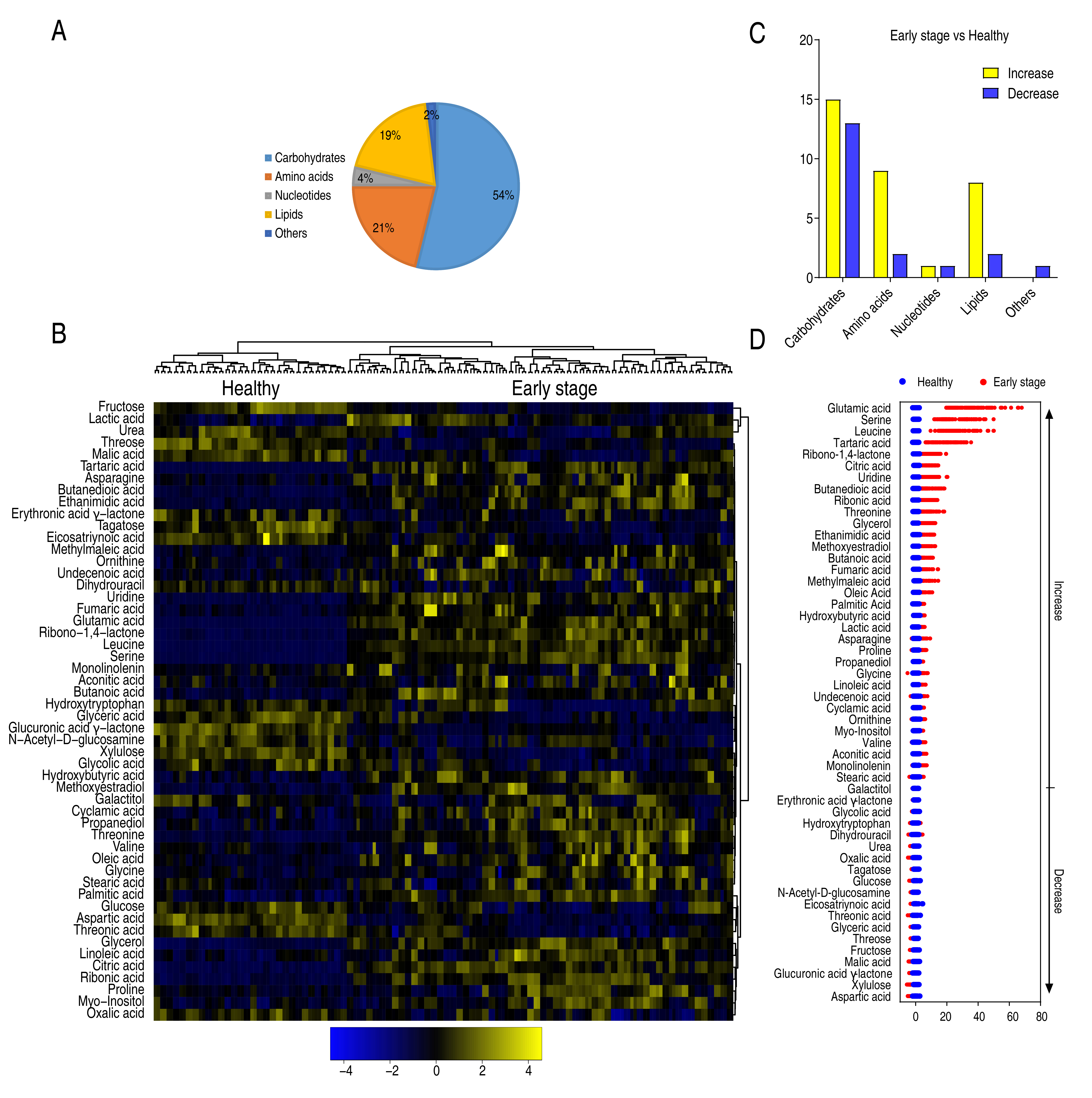

Supplement: Supplementary Figure 4 — Differential metabolomes of early-stage esophageal cancer patients. A. Categories of the differential abundance of metabolites. B. Heatmap showing differential abundance of metabolites. Yellow color and blue color indicate increase and decrease of metabolites relative to the median metabolite level, respectively (see color scale). C. Number of differentially abundant metabolites is increased and decreased in every category. D. Z-score plot of differential metabolites based on control (healthy). [file Image_4.jpeg]

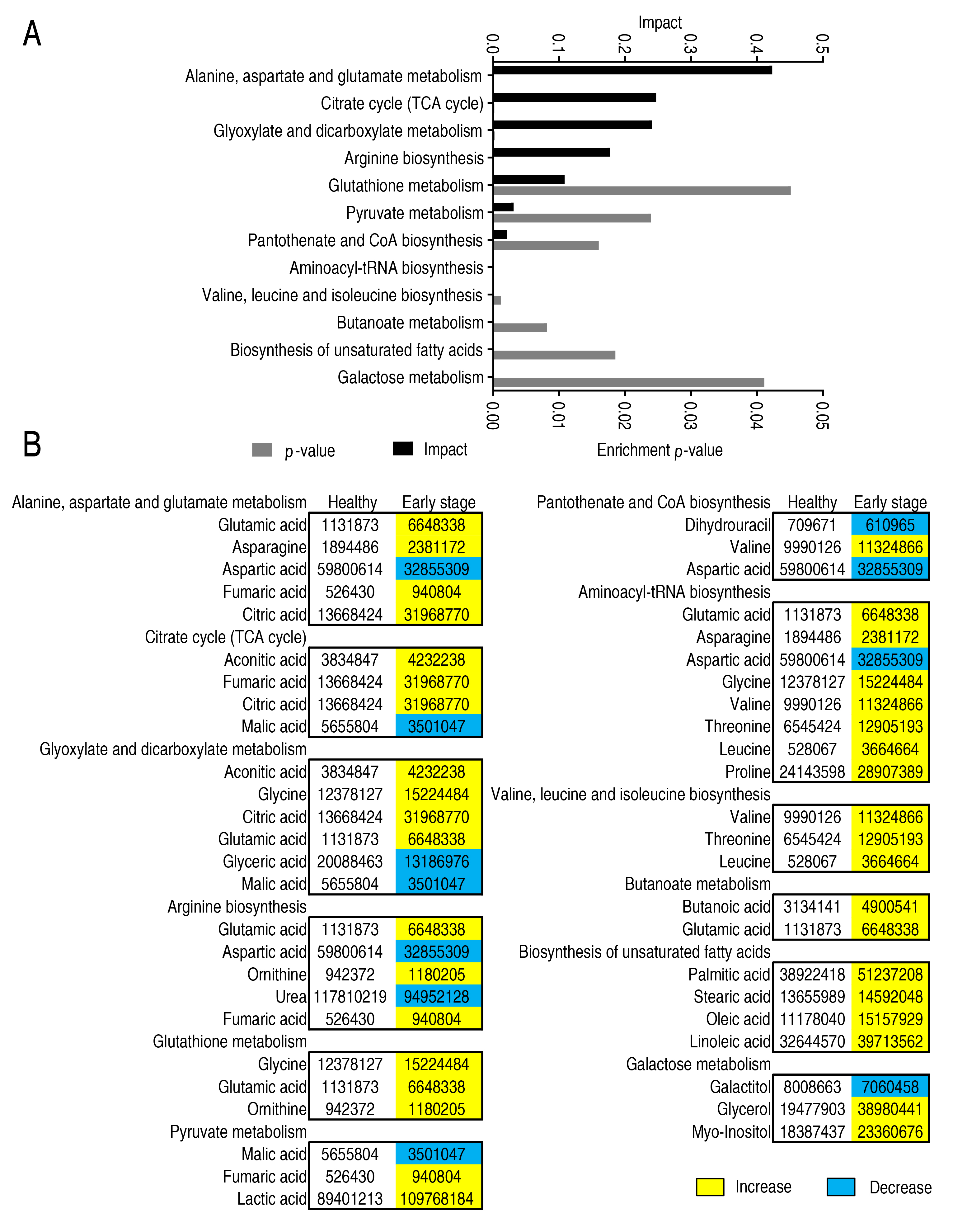

Supplement: Supplementary Figure 5 — Pathway analysis of early-stage esophageal cancer patients. A. Pathway enrichment of differential abundant metabolites. B. The levels of the differential metabolites. [file Image_5.jpeg]

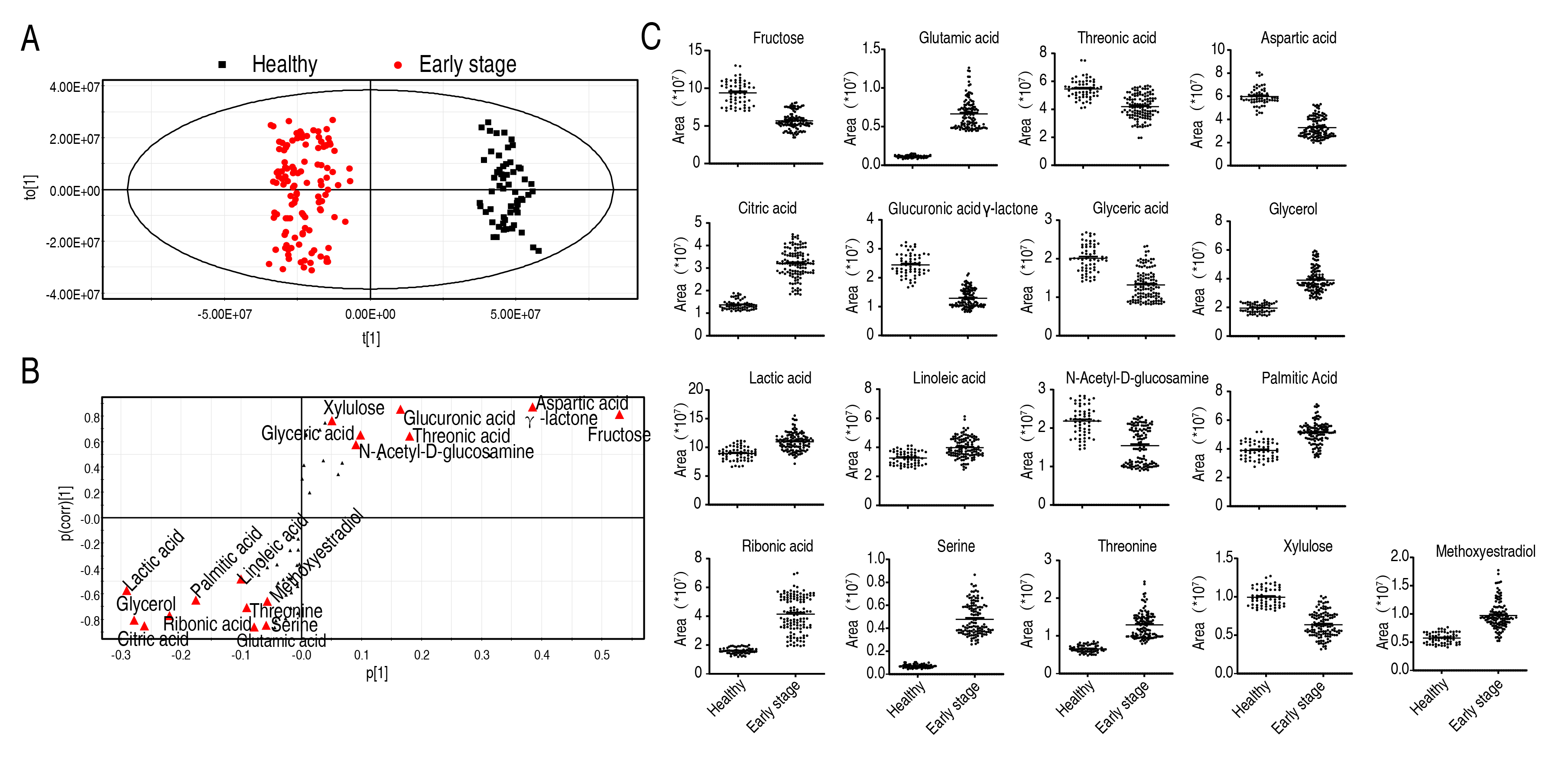

Supplement: Supplementary Figure 6 — Identification for biomarkers in patients with early-stage esophageal cancer. A. OPLS-DA of healthy group and early-stage esophageal cancer group. B. S-plot is generated from OPLS-DA. C. Abundance of biomarkers. Results (C) are displayed as mean ± SEM. [file Image_6.jpeg]

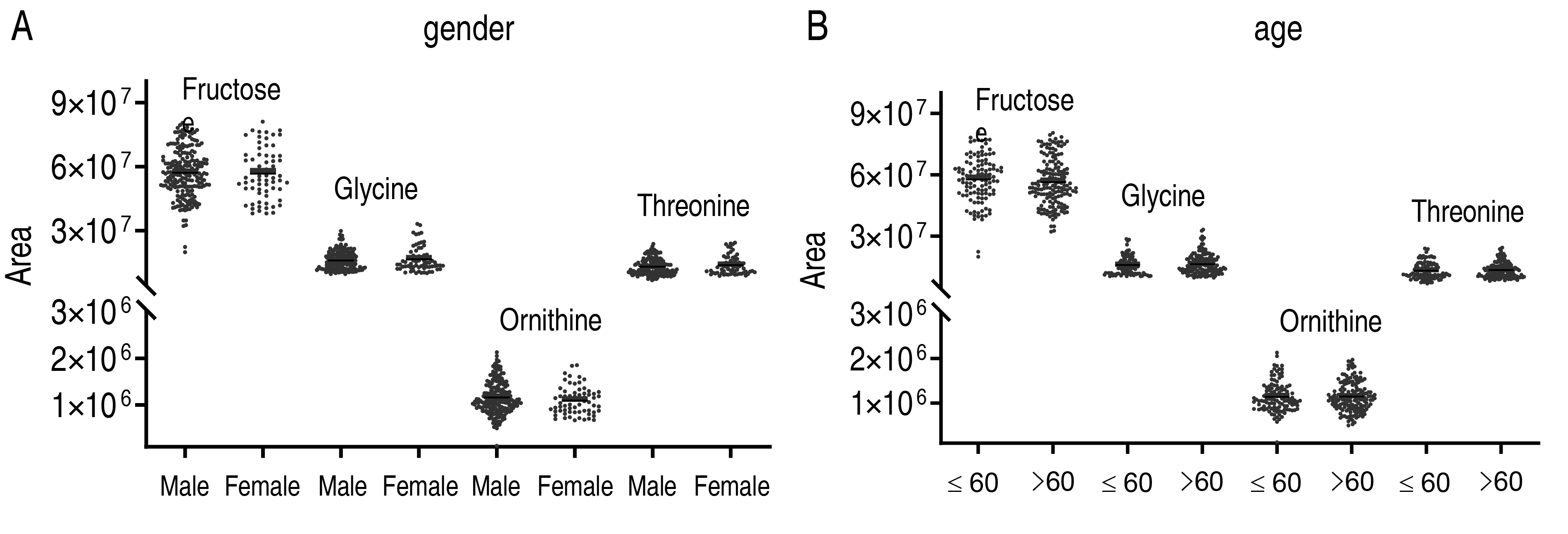

Supplement: Supplementary Figure 7 — Levels of metabolites in esophageal cancer patients with different gender and age. A. Levels of fructose, glycine, ornithine and threonine in patients with esophageal cancer of different genders. B. Abundance of fructose, glycine, ornithine and threonine in patients with esophageal cancer at different age groups (≤ 60 or > 60). [file Image_7.jpeg]
