# Supplementary material for: Untargeted serum metabolomics reveals potential biomarkers and metabolic pathways associated with esophageal cancer
Source: Front Oncol. 2022 Sep 13;12:938234. doi: 10.3389/fonc.2022.938234 (PMC9513043; doi:10.3389/fonc.2022.938234)
Supplement: Supplementary file 9 [file DataSheet_2.docx]

**Supplementary Table 2.** **Laboratory diagnosis potential of 15** **biomarkers in identifying esophageal cancer from healthy individuals**

|  | Youden  index | Cut off  value | Healthy  N. (%) | Esophageal cancer  N. (%) | Total  N. (%) |
| --- | --- | --- | --- | --- | --- |
| Glutamic acid | 1 | 2590766 | 30 (100.0) | 137 (100.0) | 167 (100.0) |
| Leucine | 1 | 1409044 | 30 (100.0) | 137 (100.0) | 167 (100.0) |
| Serine | 1 | 1414258 | 30 (100.0) | 137 (100.0) | 167 (100.0) |
| Citric acid | 0.970803 | 18925315 | 30 (100.0) | 133 (97.1) | 163 (97.6) |
| Aspartic acid | 0.911436 | 52641474 | 28 (93.3) | 134 (97.8) | 162 (97.0) |
| Ribonic acid | 0.937470 | 19297706 | 29 (96.7) | 133 (97.1) | 162 (97.0) |
| Threonine | 0.963504 | 8469026 | 30 (100.0) | 132 (96.4) | 162 (97.0) |
| Glycerol | 0.956204 | 24515640 | 30 (100.0) | 131 (95.6) | 161 (96.4) |
| Glucuronic acid γ-lactone | 0.900973 | 18651280 | 29 (96.7) | 128 (93.4) | 157 (94.0) |
| Xylulose | 0.890511 | 8255444 | 30 (100.0) | 122 (89.1) | 152 (91.0) |
| Fructose | 0.846715 | 70114711 | 30 (100.0) | 116 (84.7) | 146 (87.4) |
| Threonic acid | 0.698783 | 48453101 | 26 (86.7) | 114 (83.2) | 140 (83.8) |
| Palmitic acid | 0.754988 | 47035412 | 29 (96.7) | 108 (78.8) | 137 (82.0) |
| Glyceric acid | 0.759124 | 14153554 | 30 (100.0) | 104 (75.9) | 134 (80.2) |
| Linoleic acid | 0.492214 | 39218276 | 29 (96.7) | 72 (52.6) | 101 (60.5) |

**Supplementary Table 3.** **Laboratory diagnosis potential of glycine in identifying early-stage from late-stage esophageal cancer**

|  | Youden  index | Cut off  value | Early-stage  N. (%) | Late-stage  N. (%) | Total  N. (%) |
| --- | --- | --- | --- | --- | --- |
| Glycine | 0.374468 | 18336207 | 48 (80.0) | 27 (57.4) | 75 (70.1) |

**Supplementary Table 4.** **Laboratory diagnosis potential of 4** **biomarkers in identifying patients who received treatment from those who did not receive treatment**

|  | Youden  index | Cut off  value | Pre-treatment  N. (%) | Post-treatment  N. (%) | Total  N. (%) |
| --- | --- | --- | --- | --- | --- |
| Glycine | 0.411838 | 11956896 | 94 (87.9) | 16 (53.3) | 110 (80.3) |
| Ornithine | 0.399377 | 1121225 | 57 (53.3) | 26 (86.7) | 83 (60.6) |
| Threonine | 0.251402 | 12704790 | 59 (55.1) | 21 (70.0) | 80 (58.4) |
| Fructose | 0.347352 | 54146442 | 55 (51.4) | 25 (83.3) | 80 (58.4) |
| Glycine+Fructose |  |  | 98 (91.6) | 21 (70.0) | 119 (86.9) |
| Glycine+Ornithine |  |  | 96 (89.7) | 23 (76.7) | 119 (86.9) |
| Glycine+Threonine |  |  | 96 (89.7) | 20 (66.7) | 116 (84.7) |
| Glycine+Fructose+Ornithine |  |  | 100 (93.5) | 25 (83.3) | 125 (91.2) |
| Glycine+Fructose+Threonine |  |  | 99 (92.5) | 24 (80.0) | 123 (89.9) |
| Glycine+Fructose+Threonine+Ornithine |  |  | 101 (94.4) | 30 (100.0) | 131 (95.6) |
